# HFE-Related Hemochromatosis in a Chinese Patient: The First Reported Case

**DOI:** 10.3389/fgene.2020.00077

**Published:** 2020-02-21

**Authors:** Wei Zhang, Xiaoming Wang, Weijia Duan, Anjian Xu, Xinyan Zhao, Jian Huang, Hong You, Pierre Brissot, Xiaojuan Ou, Jidong Jia

**Affiliations:** ^1^ Liver Research Center, Beijing Friendship Hospital, Capital Medical University, Beijing Key Laboratory of Translational Medicine on Liver Cirrhosis, Beijing, China; ^2^ Clinical Research Center for Rare Liver Diseases, Capital Medical University, Beijing, China; ^3^ Liver Research Center, National Clinical Research Center for Digestive Diseases, Beijing, China; ^4^ Experimental Center, Beijing Friendship Hospital, Capital Medical University, Beijing, China; ^5^ Institut NuMeCan, InsermU-1241, University of Rennes1, Rennes, France

**Keywords:** *HFE*, compound heterozygosity, hemochromatosis, iron overload, Chinese

## Abstract

*HFE*-related Hemochromatosis is the most common genetic iron overload disease in European populations, particularly of Nordic or Celtic ancestry. It is reported that the *HFE* p.C282Y mutation is present in 1/10 people of northern European descent, resulting in one in two hundred people will be homozygous. However, the *HFE* p.C282Y heterozygosity is virtually absent among East Asians, including Japanese, Koreans, and Chinese. In this article, we report a case of *HFE*-related hemochromatosis caused by compound heterozygosity *HFE* p.C282Y/p.R71X. This is the first report of hemochromatosis associated with *HFE* p.C282Y mutation in China.

## Background

*HFE*-related hemochromatosis (HH) is the most common genetic iron overload disease in European populations, particularly of Nordic or Celtic origin (Powell et al., 2016; Brissot et al., 2018). The most common mutation in the *HFE* gene is p.C282Y. It is reported that the *HFE* p.C282Y mutation is present in 1/10 people of northern European descent, resulting in one in two hundred people that will be homozygous (Powell et al., 2016). However, the *HFE* p.C282Y mutation is virtually absent among East Asians, including Japanese (Ikuta et al., 2017), Koreans (Choi et al., 2002), and Chinese (Lin et al., 2007). To our knowledge, only one case of hemochromatosis associated with *HFE* p.C282Y mutation has been reported in Japan (Sohda et al., 2001).

In this article, we report a case of hereditary hemochromatosis caused by compound heterozygosity *HFE* p.C282Y/p.R71X. This is the first report of hemochromatosis associated with *HFE* p.C282Y mutation in China.

## Case Presentation

**Figure 1:**
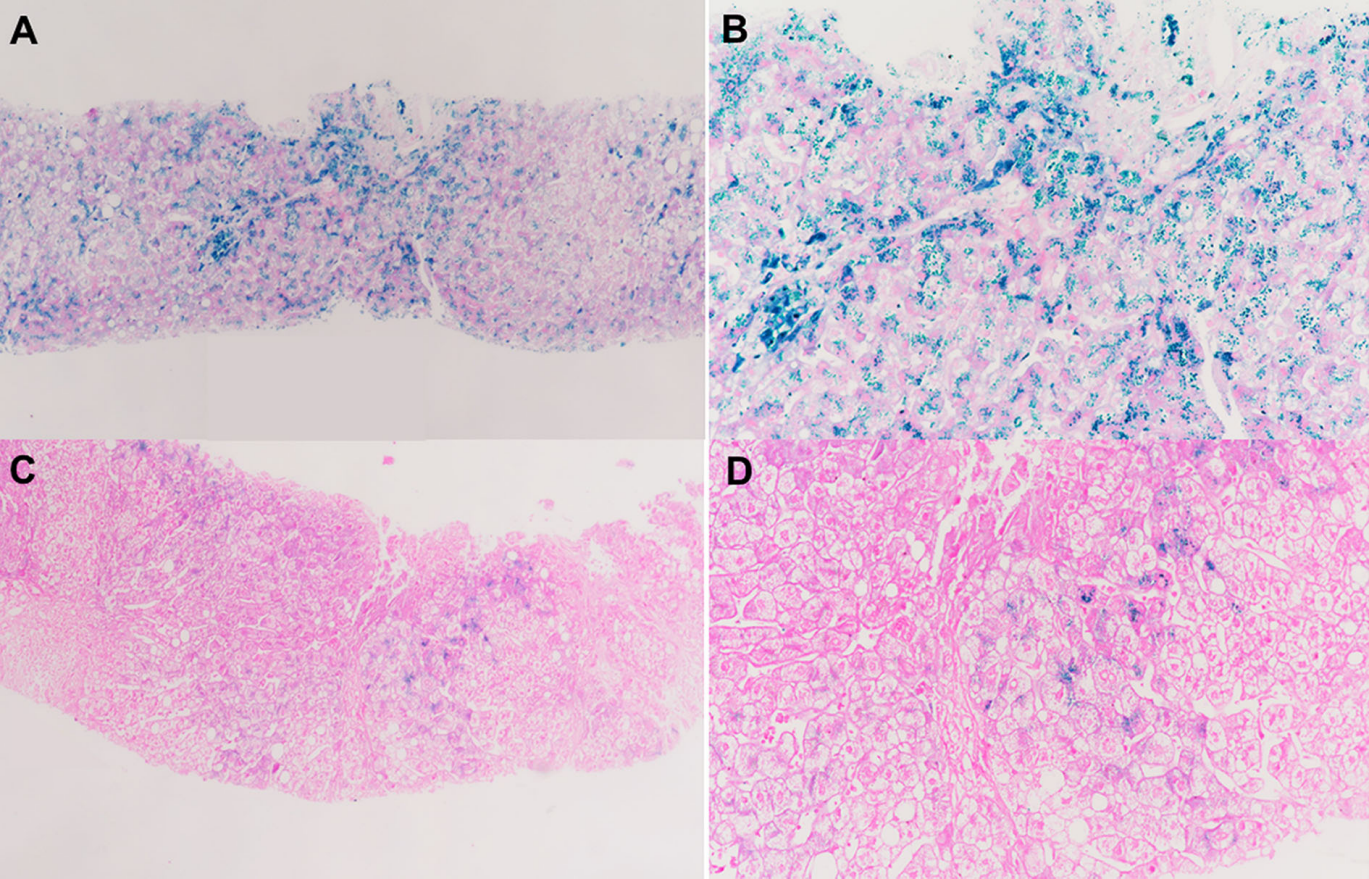
Liver biopsy data. **(A, B)** Liver biopsy of the hemochromatosis patient associated with *HFE* p.C282Y/p.R71X. It shows heavy iron accumulation in hepatocytes with little Kupffer cell siderosis. **(C, D)** Liver biopsy of the patient's father with alcoholic liver disease showing sparse iron deposits in hepatocytes. **(A, C)** Perls, 20× magnification; **(B, D)** Perls, 40× magnification.

A 28-year-old male was admitted to our department because of lasting abnormal liver function tests dating back eight years, without any clinical symptoms. He denied taking supplemental iron or consuming excess alcohol. His BMI was 24.6 kg/m^2^ and his physical examination was normal. Liver biochemistry results were as follows: alanine aminotransferase (ALT), 106 IU/L (normal: 5–40 IU/L); aspartate aminotransferase (AST), 41 IU/L (normal: 8–40 IU/L); alkaline phosphatase (ALP), 77 U/L(normal: 40–150U/L); gamma-glutamyltransferase (GGT), 19 IU/L (normal: 11–50 IU/L); and normal total and conjugated bilirubin (TB/DB), 8.8 and 3.4 µmol/L, respectively. His fasting glucose: 4.64mmol/L. Lipid profile: total cholesterol, 4mmol/L (normal: 3.90–5.20); triglyceride, 1.8 mmol/L (normal: 0.57–1.70); low density lipoprotein cholesterol, 2.30 mmol/L (normal: 2.34–3.12). Uric acid, 351.1µmol/L (normal: 178–416). Serum markers for viral hepatitis and autoimmune liver diseases were negative. Plasma iron indices: iron 38 µmol/L (normal: < 25 µmol/L), transferrin saturation 86.4% (normal: < 45%), and ferritin 3033ng/ml (normal: < 300 ng/ml). Magnetic resonance imaging (MRI) of the abdomen showed that the liver T2* value was 1.16 ms, which means severe iron overload in the liver, and the spleen was devoid of any iron overload. Liver biopsy showed severe iron deposited predominantly in hepatocytes with a decreasing gradient from periportal zone 1 to centrilobular zone 3, and iron deposits in few Kupffer cells (**Figures 1A, B**). In addition, steatosis of hepatocytes was observed, and there was mild fibrosis with stage S1.

Genetic analysis was performed in the patient and his parents to determine whether the iron overload was related to hemochromatosis. Informed consent was obtained before taking a blood sample for gene analysis of *HFE*, *HJV*, *HAMP*, *TFR2*, and *SLC40A1*. Genomic DNA was extracted from peripheral blood leukocytes using the Genomic DNA Purification Kit (Qiagen, Valencia, CA). All exons of *HFE*, *HJV, HAMP*, *TFR2*, and *SLC40A1* were PCR amplified with their associated boundary regions using the primers described in our previous study (Lv et al., 2018). PCR products were sequenced in forward and reverse orientations using an automated ABI3730 DNA sequencer (ABI).

**Figure 2:**
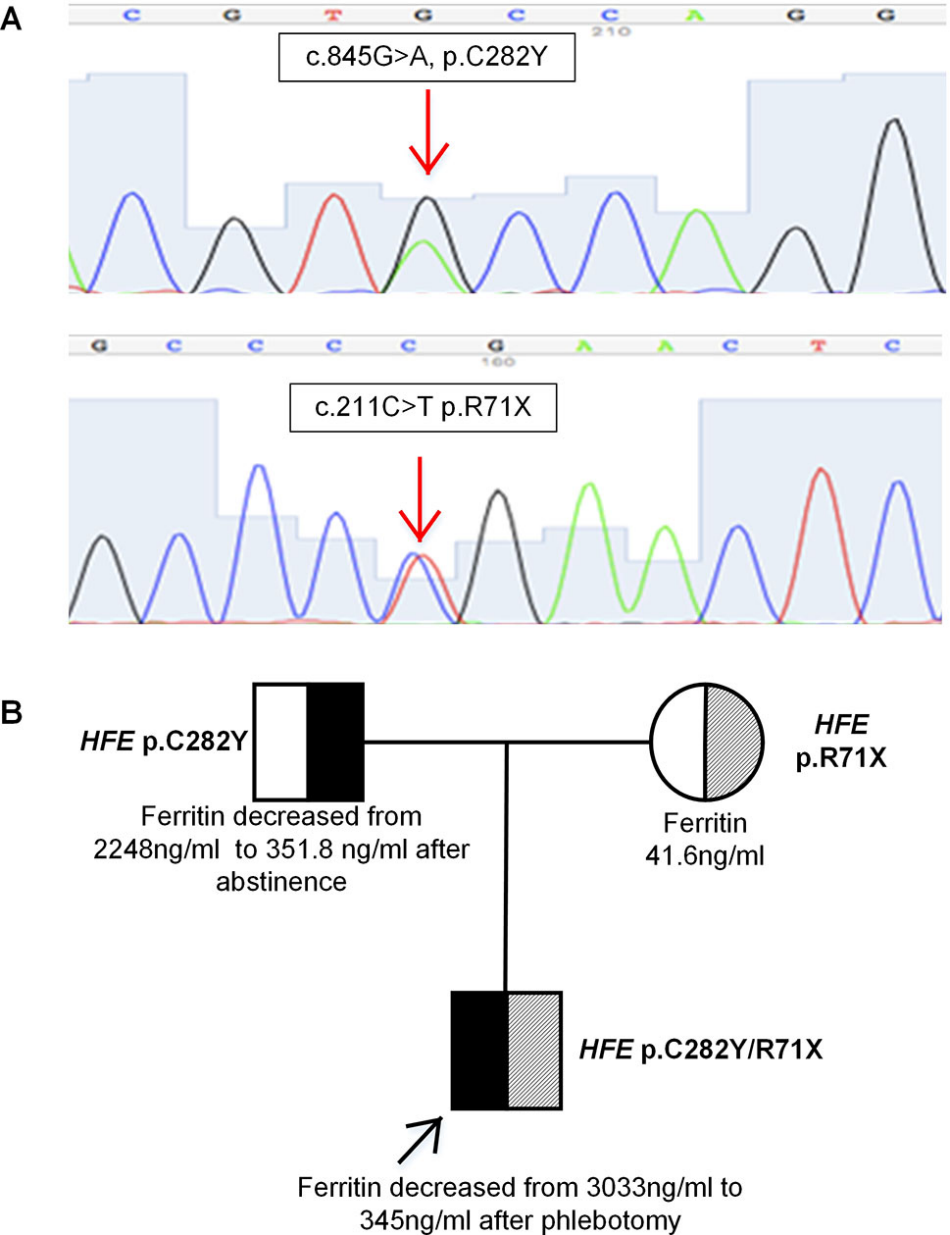
**(A)** Sequence analysis of the *HFE* gene showing two heterozygous mutations p.C282Y and p.R71X. **(B)** Pedigree analysis of the family. The patient had compound heterozygous mutations p.C282Y/p.R71X in *HFE* gene on different alleles, which were inherited from his father and mother, respectively.

The results showed that the patient had compound heterozygous mutations p.C282Y/p.R71X in the *HFE* gene (**Figure 2A**). No mutations were identified in the other four genes. His father and mother carried a heterozygous mutation p.C282Y, p.R71X in the *HFE* gene, respectively. *HFE* p.R71X has been reported in a previous study (Beutler et al., 2002). *HFE* p.R71X is a C-to-T nonsense mutation at cDNA position 211 of the *HFE* gene, resulting in the substitution of a termination codon for arginine 71 in exon 2 of the *HFE* gene.

The patient was found to be a compound heterozygote p.C282Y/p.R71X, which was inherited from his father and mother, respectively. His phenotype was typical of *HFE*-related hemochromatosis. His mother carried a heterozygous mutation p.R71X in *HFE* gene, with normal plasma liver biochemical tests and iron parameters. His father, who carried a heterozygous mutation p.C282Y in *HFE* gene, presented some degree of iron overload that could be related to alcoholic liver cirrhosis (liver biopsy is shown in **Figures 1C, D**). The segregation analysis is shown in **Figure 2B**. It should be noted that the ancestry of the patient was Chinese for several generations.

According to therapeutic recommendations released by Hemochromatosis International (Adams et al., 2018), a therapeutic phlebotomy (400 ml of whole blood) was performed every 1–2 weeks. After 32 phlebotomies within one year and two months, 11.8 L of whole blood were withdrawn from the patient. AST and ALT returned to normal range, and plasma ferritin level decreased to 28 ng/ml. His father's plasma iron parameters decreased significantly after abstinence from alcohol. Clinical data of the patient and his father are summarized in **Table 1**.

**Table 1:** Clinical characteristics and the patient with HH and his father with secondary iron overload.

	The patient	The patient's father
Lifestyle habits	None	History of heavy drinking
Gender	Male	Male
ALT (U/L)	106	62.9
AST (U/L)	41	72.8
GGT (U/L)	19	96.8
Ferritin (ng/ml)	3,033	2,248
Skin pigmentation	None	None
Cardiac disorders	None	None
Liver histology		
-fibrosis	None	S4
-iron overload	Heavy iron deposits in hepatocytes with a decreasing gradient from zone I to III.	Mild sparse iron deposits in hepatocytes
Effective therapy	Phlebotomy	Total abstinence from alcohol
Genetic background	*HFE* p.C282Y/R71X	*HFE* p.C282Y
Final diagnosis	*HFE* related (or type 1) hereditary haemochromatosis	Secondary iron overload syndromes related to alcohol related liver injury

ALT, alanine aminotransferase; AST, aspartate aminotransferase; GGT, glutamyltranspetidase.

## Discussion

Here we report a case of hemochromatosis associated with compound heterozygous mutation p.C282Y/p.R71X in *HFE* gene in China. The patient had a phenotype highly suggestive of hepcidin deficiency with very high plasma transferrin saturation and severe hepatocyte iron overload on liver biopsy. The genotypic study confirmed the diagnosis of *HFE*-related hemochromatosis (or hemochromatosis type 1). It should be noticed that one cannot exclude that part of hyperferritinemia was related to a dysmetabolic component since this patient presented some degree of steatosis (Lorcerie et al., 2017).

This case highlighted three important clinical issues. Firstly, this is the first case of *HFE-*related hemochromatosis in a Chinese patient. It is well known that *HFE* p.C282Y is very common and responsible for most cases of hemochromatosis in Caucasians (Kowdley et al., 2019). However, *HFE* p.C282Y has been rarely identified in East Asians in previous studies (Choi et al., 2002; Lin et al., 2007; Ikuta et al., 2017). Furthermore, *HFE* p.C282Y mutation had been identified neither in a variety of disorders in China such as chronic hepatitis C, nonalcoholic fatty liver disease (Lin et al., 2005), cardiovascular disease (Bi et al., 2013), myelodysplastic syndromes, and aplastic anemia(Nie et al., 2010), nor in general population(Lin et al., 2007). In addition, we recently analyzed 22 patients with primary iron overload and no *HFE* variants were identified (Lv et al., 2018). Therefore, although very rare, *HFE* gene mutations should not be ignored as a possible cause of hemochromatosis in Asians.

The second clinical comment concerns the autosomal recessive nature of *HFE-*related hemochromatosis, so that a heterozygous mutation in *HFE* gene will not lead to hemochromatosis. It has been reported that among *HFE*-related hemochromatosis, p.C282Y homozygotes accounts for 95%, p.C282Y/H63D compound heterozygotes and p.H63D homozygotes accounts for 4%, 1%, respectively (Adams, 2015). However, the responsibility of the latter two genotypes in causing hemochromatosis remains highly debated (Porto et al., 2016). In the hemochromatosis patient here reported, the p.C282Y mutation was found in combination with a nonsense mutation R71X in *HFE* gene. *HFE* R71X mutation, resulting in the substitution of a termination codon for arginine 71 in exon 2 of the *HFE* gene, exerts marked effect on iron homeostasis, as reported in a previous study (Beutler et al., 2002). While the fact that the patient's mother has the heterozygous p.R71X mutation in the *HFE* gene but is not iron overloaded is clearly consistent with the hypothesis that this single heterozygous *HFE* mutation alone is not sufficient for iron overload, and it is just a single case.

The third comment relates to the importance of considering both the clinical history and the response to therapy for differentiating hemochromatosis from secondary iron overload. In this study, although both son and father carried a single heterozygous mutation *HFE* p.C282Y, alcohol turned out to be a key factor in causing increased plasma iron indices in the patient's father since after four months of total abstinence both liver function tests and iron parameters returned to normal. In contrast, our hemochromatosis patient did not have any excessive alcohol consumption and the sole phlebotomy therapy was able to restore normal iron parameters. It is also noteworthy that both son and father were heterozygous for the p.C282Y mutation: this genetic profile could not, a priori, be considered as responsible for iron overload since the vast majority of *HFE*-related hemochromatosis cases are related to p.C282Y homozygosity. It is precisely the peculiar phenotypic profile of the son which justified pursuing the genetic investigations leading to the finding of another type (this time deleterious) of *HFE*-related compound heterozygosity. Finally, it should be noted that the phenotypic profile of our patient was close to that described as “juvenile” hemochromatosis (severe iron overload before the age of 30 years).

## Conclusion

*HFE*-related hemochromatosis was identified in a Chinese hemochromatosis patient for the first time. Although very rare in Asians, this diagnostic possibility should not be ignored, given its major importance for the proband him(her)self and for his (her) family. In practice, clinicians should use a combination of phenotypic and genotypic information to suggest and confirm the diagnosis of hemochromatosis.

## Data Availability Statement

The raw data supporting the conclusions of this article will be made available by the authors, without undue reservation, to any qualified researcher.

## Ethics Statement

Written informed consent to publish this case report was obtained from the patient and his family.

## Author Contributions

WZ made substantial contributions to conception and design, acquisition, analysis, and interpretation of the data, and involved in drafting the manuscript. XW, WD, AX, XZ, and HY made substantial contributions to the acquisition, analysis, and interpretation of the data. JH and PB involved in critically revising the manuscript for important intellectual content. XO and JJ made substantial contributions to conception and design and involved in revising the manuscript critically for important intellectual content, and gave the final approval of the version to be published.

## Funding

This study was supported by grants from The Beijing Natural Science Foundation (No.7194254) and The Digestive Medical Coordinated Development Center of Beijing Hospitals Authority (No. XXZ0502).

## Conflict of Interest

The authors declare that the research was conducted in the absence of any commercial or financial relationships that could be construed as a potential conflict of interest.
